# Supplementary material for: Unveiling the Bioactive Potential of the Invasive Jellyfish Phyllorhiza punctata Through Integrative Transcriptomic and Proteomic Analyses
Source: Biomolecules. 2025 Aug 4;15(8):1121. doi: 10.3390/biom15081121 (PMC12383608; doi:10.3390/biom15081121)
Supplement: Supplementary file 1 [file biomolecules-15-01121-s001.zip › Suplementary Material/FileS3/PEPFOLD4/XP_065051697_13335/clusters.html]

 PEP-FOLD clusters


Download archive of all models  here   

|  |  |  |
| --- | --- | --- |
| # | file | sOPEP |
| # cluster 1 - Size: 2 |  |  |
|  | XP\_065051697\_13335-model1 | -30.8769 |
|  | XP\_065051697\_13335-model1.1 | -29.5466 |
| # cluster 2 - Size: 1 |  |  |
|  | XP\_065051697\_13335-model2 | -30.2829 |
| # cluster 3 - Size: 3 |  |  |
|  | XP\_065051697\_13335-model3 | -29.9283 |
|  | XP\_065051697\_13335-model3.1 | -27.8927 |
|  | XP\_065051697\_13335-model3.2 | -26.8598 |
| # cluster 4 - Size: 2 |  |  |
|  | XP\_065051697\_13335-model4 | -28.5347 |
|  | XP\_065051697\_13335-model4.1 | -24.8162 |
| # cluster 5 - Size: 1 |  |  |
|  | XP\_065051697\_13335-model5 | -28.3149 |
| # cluster 6 - Size: 2 |  |  |
|  | XP\_065051697\_13335-model6 | -28.0492 |
|  | XP\_065051697\_13335-model6.1 | -26.9088 |
| # cluster 7 - Size: 5 |  |  |
|  | XP\_065051697\_13335-model7 | -28.0095 |
|  | XP\_065051697\_13335-model7.1 | -27.0714 |
|  | XP\_065051697\_13335-model7.2 | -26.6376 |
|  | XP\_065051697\_13335-model7.3 | -24.4575 |
|  | XP\_065051697\_13335-model7.4 | -21.127 |
| # cluster 8 - Size: 2 |  |  |
|  | XP\_065051697\_13335-model8 | -27.806 |
|  | XP\_065051697\_13335-model8.1 | -22.6678 |
| # cluster 9 - Size: 2 |  |  |
|  | XP\_065051697\_13335-model9 | -27.6275 |
|  | XP\_065051697\_13335-model9.1 | -22.8703 |
| # cluster 10 - Size: 2 |  |  |
|  | XP\_065051697\_13335-model10 | -26.9954 |
|  | XP\_065051697\_13335-model10.1 | -24.3694 |
| # cluster 11 - Size: 2 |  |  |
|  | XP\_065051697\_13335-model11 | -26.1455 |
|  | XP\_065051697\_13335-model11.1 | -24.3794 |
| # cluster 12 - Size: 1 |  |  |
|  | XP\_065051697\_13335-model12 | -25.5426 |
| # cluster 13 - Size: 1 |  |  |
|  | XP\_065051697\_13335-model13 | -25.516 |
| # cluster 14 - Size: 3 |  |  |
|  | XP\_065051697\_13335-model14 | -25.434 |
|  | XP\_065051697\_13335-model14.1 | -25.3575 |
|  | XP\_065051697\_13335-model14.2 | -23.0883 |
| # cluster 15 - Size: 6 |  |  |
|  | XP\_065051697\_13335-model15 | -25.3802 |
|  | XP\_065051697\_13335-model15.1 | -24.9689 |
|  | XP\_065051697\_13335-model15.2 | -21.8982 |
|  | XP\_065051697\_13335-model15.3 | -21.3758 |
|  | XP\_065051697\_13335-model15.4 | -21.2534 |
|  | XP\_065051697\_13335-model15.5 | -19.8508 |
| # cluster 16 - Size: 3 |  |  |
|  | XP\_065051697\_13335-model16 | -25.2092 |
|  | XP\_065051697\_13335-model16.1 | -23.1646 |
|  | XP\_065051697\_13335-model16.2 | -15.0078 |
| # cluster 17 - Size: 2 |  |  |
|  | XP\_065051697\_13335-model17 | -24.6693 |
|  | XP\_065051697\_13335-model17.1 | -18.5031 |
| # cluster 18 - Size: 1 |  |  |
|  | XP\_065051697\_13335-model18 | -24.3032 |
| # cluster 19 - Size: 3 |  |  |
|  | XP\_065051697\_13335-model19 | -24.0406 |
|  | XP\_065051697\_13335-model19.1 | -16.7065 |
|  | XP\_065051697\_13335-model19.2 | -15.8436 |
| # cluster 20 - Size: 1 |  |  |
|  | XP\_065051697\_13335-model20 | -23.9896 |
| # cluster 21 - Size: 1 |  |  |
|  | XP\_065051697\_13335-model21 | -23.4004 |
| # cluster 22 - Size: 2 |  |  |
|  | XP\_065051697\_13335-model22 | -23.0905 |
|  | XP\_065051697\_13335-model22.1 | -21.494 |
| # cluster 23 - Size: 1 |  |  |
|  | XP\_065051697\_13335-model23 | -23.0842 |
| # cluster 24 - Size: 1 |  |  |
|  | XP\_065051697\_13335-model24 | -22.8523 |
| # cluster 25 - Size: 3 |  |  |
|  | XP\_065051697\_13335-model25 | -22.7202 |
|  | XP\_065051697\_13335-model25.1 | -20.4687 |
|  | XP\_065051697\_13335-model25.2 | -16.5108 |
| # cluster 26 - Size: 3 |  |  |
|  | XP\_065051697\_13335-model26 | -21.9865 |
|  | XP\_065051697\_13335-model26.1 | -21.4379 |
|  | XP\_065051697\_13335-model26.2 | -20.2504 |
| # cluster 27 - Size: 1 |  |  |
|  | XP\_065051697\_13335-model27 | -21.9852 |
| # cluster 28 - Size: 2 |  |  |
|  | XP\_065051697\_13335-model28 | -21.953 |
|  | XP\_065051697\_13335-model28.1 | -21.2477 |
| # cluster 29 - Size: 2 |  |  |
|  | XP\_065051697\_13335-model29 | -21.6723 |
|  | XP\_065051697\_13335-model29.1 | -20.0453 |
| # cluster 30 - Size: 3 |  |  |
|  | XP\_065051697\_13335-model30 | -21.459 |
|  | XP\_065051697\_13335-model30.1 | -21.0489 |
|  | XP\_065051697\_13335-model30.2 | -16.751 |
| # cluster 31 - Size: 2 |  |  |
|  | XP\_065051697\_13335-model31 | -21.2889 |
|  | XP\_065051697\_13335-model31.1 | -17.8359 |
| # cluster 32 - Size: 1 |  |  |
|  | XP\_065051697\_13335-model32 | -21.1897 |
| # cluster 33 - Size: 1 |  |  |
|  | XP\_065051697\_13335-model33 | -20.909 |
| # cluster 34 - Size: 2 |  |  |
|  | XP\_065051697\_13335-model34 | -20.7854 |
|  | XP\_065051697\_13335-model34.1 | -19.6808 |
| # cluster 35 - Size: 1 |  |  |
|  | XP\_065051697\_13335-model35 | -20.7621 |
| # cluster 36 - Size: 1 |  |  |
|  | XP\_065051697\_13335-model36 | -20.7457 |
| # cluster 37 - Size: 1 |  |  |
|  | XP\_065051697\_13335-model37 | -20.7272 |
| # cluster 38 - Size: 2 |  |  |
|  | XP\_065051697\_13335-model38 | -20.5591 |
|  | XP\_065051697\_13335-model38.1 | -13.9586 |
| # cluster 39 - Size: 2 |  |  |
|  | XP\_065051697\_13335-model39 | -20.1233 |
|  | XP\_065051697\_13335-model39.1 | -17.3102 |
| # cluster 40 - Size: 1 |  |  |
|  | XP\_065051697\_13335-model40 | -18.934 |
| # cluster 41 - Size: 1 |  |  |
|  | XP\_065051697\_13335-model41 | -18.8055 |
| # cluster 42 - Size: 3 |  |  |
|  | XP\_065051697\_13335-model42 | -18.6089 |
|  | XP\_065051697\_13335-model42.1 | -18.5691 |
|  | XP\_065051697\_13335-model42.2 | -17.2997 |
| # cluster 43 - Size: 2 |  |  |
|  | XP\_065051697\_13335-model43 | -18.5569 |
|  | XP\_065051697\_13335-model43.1 | -17.9935 |
| # cluster 44 - Size: 1 |  |  |
|  | XP\_065051697\_13335-model44 | -17.4545 |
| # cluster 45 - Size: 1 |  |  |
|  | XP\_065051697\_13335-model45 | -17.4432 |
| # cluster 46 - Size: 1 |  |  |
|  | XP\_065051697\_13335-model46 | -17.3274 |
| # cluster 47 - Size: 1 |  |  |
|  | XP\_065051697\_13335-model47 | -17.164 |
| # cluster 48 - Size: 1 |  |  |
|  | XP\_065051697\_13335-model48 | -17.1248 |
| # cluster 49 - Size: 1 |  |  |
|  | XP\_065051697\_13335-model49 | -17.1067 |
| # cluster 50 - Size: 1 |  |  |
|  | XP\_065051697\_13335-model50 | -16.8893 |
| # cluster 51 - Size: 1 |  |  |
|  | XP\_065051697\_13335-model51 | -16.7294 |
| # cluster 52 - Size: 1 |  |  |
|  | XP\_065051697\_13335-model52 | -16.3453 |
| # cluster 53 - Size: 1 |  |  |
|  | XP\_065051697\_13335-model53 | -15.8411 |
| # cluster 54 - Size: 2 |  |  |
|  | XP\_065051697\_13335-model54 | -15.7279 |
|  | XP\_065051697\_13335-model54.1 | -15.0128 |
| # cluster 55 - Size: 1 |  |  |
|  | XP\_065051697\_13335-model55 | -15.3527 |
| # cluster 56 - Size: 1 |  |  |
|  | XP\_065051697\_13335-model56 | -14.5801 |
| # cluster 57 - Size: 1 |  |  |
|  | XP\_065051697\_13335-model57 | -14.2971 |
| # cluster 58 - Size: 1 |  |  |
|  | XP\_065051697\_13335-model58 | -14.2807 |
|  |  |  |
